# 4-[(4-Bromo­phenyl)amino]-2-methyl­idene-4-oxo­butanoic acid

**DOI:** 10.1107/S1600536814012872

**Published:** 2014-06-14

**Authors:** B. Narayana, Prakash S. Nayak, Balladka K. Sarojini, Jerry P. Jasinski

**Affiliations:** a Department of Studies in Chemistry, Mangalore University, Mangalagangotri 574 199, India; b Department of Studies in Chemistry, Industrial Chemistry Section, Mangalore University, Mangalagangotri 574 199, India; c Department of Chemistry, Keene State College, 229 Main Street, Keene, NH 03435-2001, USA

## Abstract

In the title compound, C_11_H_10_BrNO_3_, two independent mol­ecules (*A* and *B*) crystallize in the asymmetric unit. The dihedral angles between the mean planes of the 4-bromo­phenyl ring and amide group are 24.8 (7) in mol­ecule *A* and 77.1 (6)° in mol­ecule *B*. The mean plane of the methyl­idene group is further inclined by 75.6 (4) in mol­ecule *A* and 72.5 (6)° in mol­ecule *B* from that of the amide group. In the crystal, N—H⋯O hydrogen bonds formed by amide groups and O—H⋯O hydrogen bonds formed by carb­oxy­lic acid groups are observed and supported additionally by weak C—H⋯O inter­actions between the methyl­idene and amide groups. Together, these link the mol­ecules into chains of dimers along [110] and form *R*_2_^2^(8) graph-set motifs.

## Related literature   

For the pharmacological activity of amide derivatives, see: Galanakis *et al.* (2004 ▶); Kumar & Knaus (1993 ▶); Ban *et al.* (1998 ▶); Ukrainets *et al.* (2006 ▶), Lesyk & Zimenkovsky (2004 ▶); Gududuru *et al.* (2004 ▶). For related structures, see: Nayak *et al.* (2013*a* ▶,*b* ▶). For standard bond lengths, see: Allen *et al.* (1987 ▶).
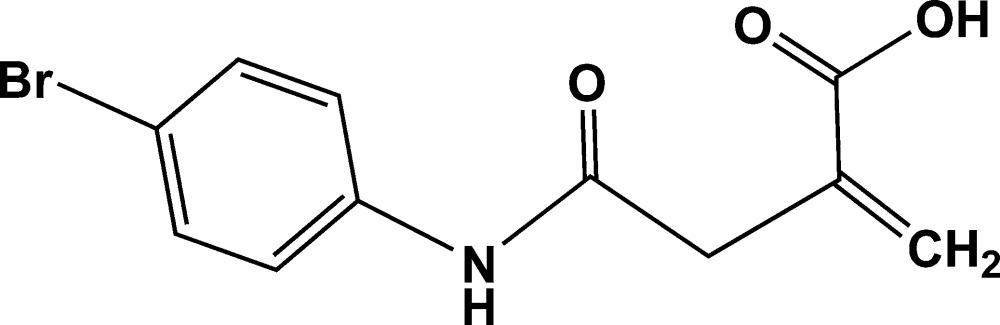


## Experimental   

### 

#### Crystal data  

C_11_H_10_BrNO_3_
*M* *_r_* = 284.11
Triclinic, 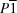

*a* = 6.2782 (4) Å
*b* = 8.3251 (5) Å
*c* = 21.3244 (12) Å
α = 96.462 (5)°
β = 92.026 (5)°
γ = 95.390 (5)°
*V* = 1101.38 (11) Å^3^
*Z* = 4
Cu *K*α radiation
μ = 5.04 mm^−1^
*T* = 173 K
0.44 × 0.28 × 0.14 mm

#### Data collection  

Agilent Eos Gemini diffractometer
Absorption correction: multi-scan (*CrysAlis PRO* and *CrysAlis RED*; Agilent, 2012 ▶) *T*_min_ = 0.162, *T*_max_ = 1.000
7163 measured reflections
4131 independent reflections
3490 reflections with *I* > 2σ(*I*)
*R*_int_ = 0.033

#### Refinement  

*R*[*F* ^2^ > 2σ(*F* ^2^)] = 0.076
*wR*(*F* ^2^) = 0.227
*S* = 1.03
4131 reflections
291 parameters
H-atom parameters constrained
Δρ_max_ = 2.73 e Å^−3^
Δρ_min_ = −0.79 e Å^−3^

### 

Data collection: *CrysAlis PRO* (Agilent, 2012 ▶); cell refinement: *CrysAlis PRO*; data reduction: *CrysAlis RED* (Agilent, 2012 ▶); program(s) used to solve structure: *SUPERFLIP* (Palatinus *et al.*, 2012 ▶); program(s) used to refine structure: *SHELXL2012* (Sheldrick, 2008 ▶); molecular graphics: *OLEX2* (Dolomanov *et al.*, 2009 ▶); software used to prepare material for publication: *OLEX2*.

## Supplementary Material

Crystal structure: contains datablock(s) I. DOI: 10.1107/S1600536814012872/bt6983sup1.cif

Structure factors: contains datablock(s) I. DOI: 10.1107/S1600536814012872/bt6983Isup2.hkl

Supporting information file. DOI: 10.1107/S1600536814012872/bt6983Isup3.cml

CCDC reference: 1006395

Additional supporting information:  crystallographic information; 3D view; checkCIF report

## Figures and Tables

**Table 1:** Hydrogen-bond geometry (Å, °)

*D*—H⋯*A*	*D*—H	H⋯*A*	*D*⋯*A*	*D*—H⋯*A*
O3*A*—H3*A*⋯O2*B* ^i^	0.84	1.85	2.685 (5)	174
N1*A*—H1*A*⋯O1*B* ^ii^	0.88	2.06	2.933 (5)	170
O3*B*—H3*B*⋯O2*A* ^iii^	0.84	1.82	2.654 (5)	170
N1*B*—H1*B*⋯O1*A* ^iv^	0.88	2.04	2.848 (6)	152
C5*B*—H5*BB*⋯O1*A* ^v^	0.95	2.54	3.464 (7)	164

Symmetry codes: (i) 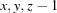; (ii) 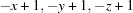; (iii) 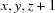; (iv) 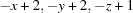; (v) 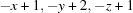.

**Fig. 1.**
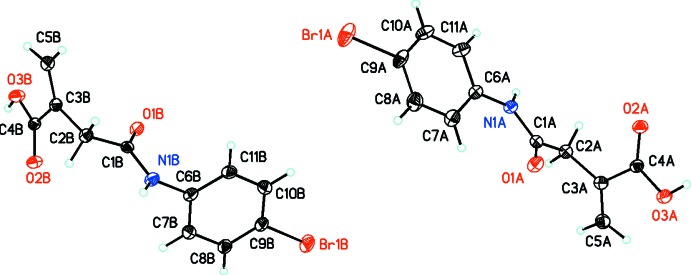
ORTEP drawing of the title compound, C11H10BrNO3, showing the labeling scheme with 30% probability displacement ellipsoids.

**Fig. 2.**
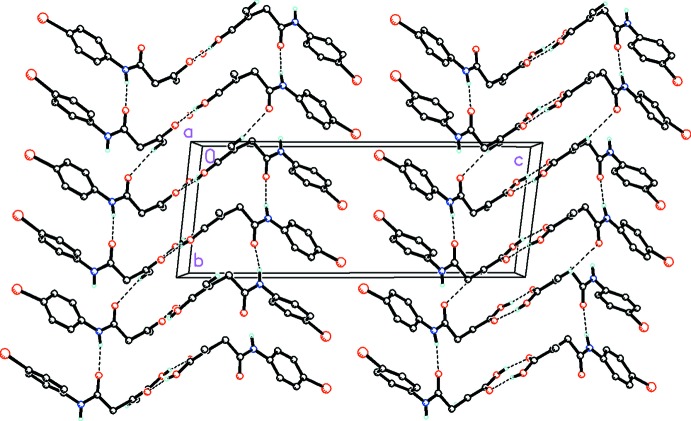
Molecular packing for the title compound viewed along the a axis. Dashed lines indicate N—H···O, O—H···O hydrogen bonds and weak C—H···O intermolecular interactions linking the molecules into chains of dimers along [1 1 0]. H atoms not involved in hydrogen bonding or weak intermolecular interactions have been removed for clarity.

**Fig. 3.**
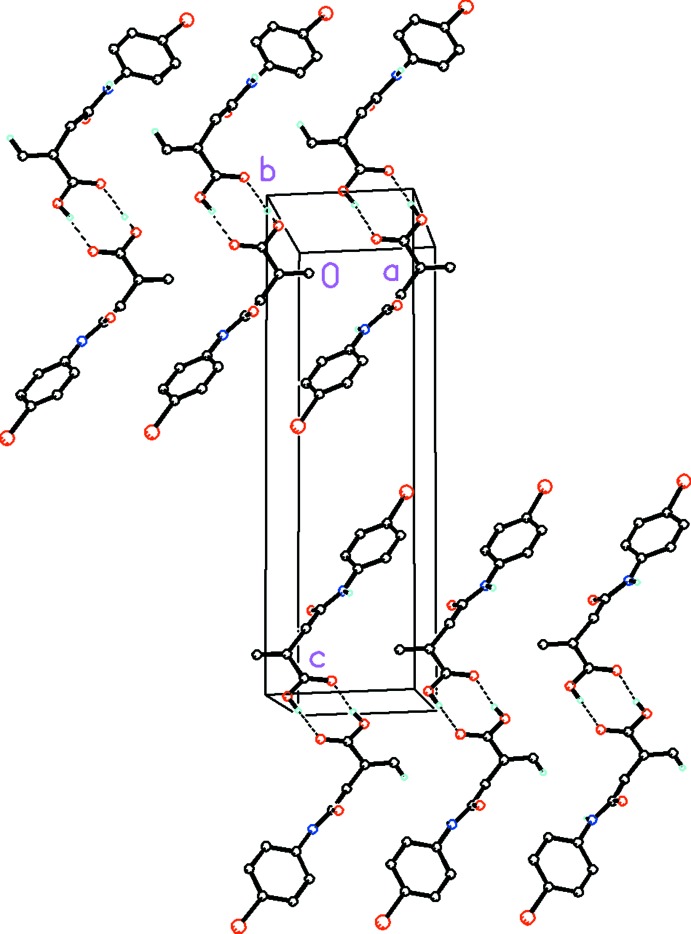
Molecular packing for the title compound viewed along the b axis. Dashed lines indicate O—H···O hydrogen bonds between the carboxylic groups forming R22(8) graph-set motifs linking the molecules into chains of dimers along [1 1 0]. H atoms not involved in hydrogen bonding have been removed for clarity.

**Fig. 4.**
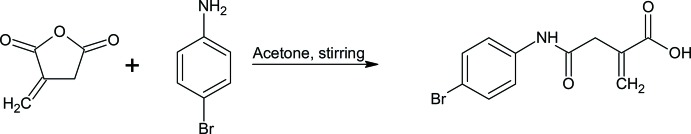
Synthesis of C11H10BrNO3.

## supplementary crystallographic information

### S1. Comment

Amide bonds play a major role in the elaboration and composition of biological systems, which are the main chemical bonds that link amino acid building blocks together to give proteins. Amide bonds are not limited to biological systems and are indeed present in a huge array of molecules, including major marketed drugs. Amide derivatives possessing anti-inflammatory (Galanakis *et al.*, 2004; Kumar *et al.*, 1993; Ban *et al.*, 1998), antimicrobial (Ukrainets *et al.*, 2006), anti-tubercular (Lesyk *et al.*, 2004) and antiproliferative (Gududuru *et al.*, 2004) activities are reported in the literature. Crystal structures of some related amide derivatives include, viz., 4-(4-iodoanilino)-2-methylene-4-oxobutanoic acid and 4-(3-fluoro-4-methylanilino)-2-methylidene-4-oxobutanoic acid (Nayak *et al.*, 2013*a*,*b*). Hence in view of its pharmacological importance, the title compound 4-[(4-bromophenyl)amino]-2-methylidene-4-oxobutanoic acid (I), C_11_H_10_BrNO_3_, was synthesized from 3-methylidenedihydrofuran-2,5-dione with good yields and its crystal structure is reported here.

In the title compound, two independent molecules (A & B) crystallize in the asymmetric unit (Fig. 1). The N–C(=O) bond lengths of 1.359 (6)A (A) and 1.346 (6)Å (B) are indicative of amide-type resonance. The bond lengths of the remaining atoms are in normal ranges (Allen *et al.*, 1987). In the crystal, classical N—H···O and O—H···O hydrogen bonds are observed supported additionally by weak C—H···O intermolecular interactions between the 2-methylidene and amide groups (Table 1, Fig. 2) linking the molecules into chains of dimers along [1 1 0]. The N—H···O hydrogen bonds are supported by the carbonyl oxygen atom of the amide functionality as the acceptor. The carboxylic acid groups form a dimeric hydrogen bonding pattern commonly seen for many carbolylic acids into *R*_2_^2^(8) graph-set motifs (Fig. 3). The dihedral angles between the mean planes of the 4-bromophenyl ring (C6A–C11A or C6B–C11B) and oxoamine group (N1A/C1A/O1A/C2A or N1B/C1B/O1B/C2B) are 24.8 (7)° (A) and 77.1 (6)° (B), respectively. The mean plane of the 2-methylidene group (C2A–C5A or C2B–C5B) is further inclined by 75.6 (4)° (A) and 72.5 (6)° (B) from that of the oxoamine group (N1A/C1A/O1A/C2A or N1B/C1B/O1B/C2B).

### S2. Experimental

3-Methylidenedihydrofuran-2,5-dione (0.112 g, 1 mmol) was dissolved in 30 ml acetone and stirred at ambient temperature. 4-Bromoaniline (0.172 g, 1 mmol) in 20 mL acetone was added over 30 mins (Fig. 4). After sirring for 1.5 h the slurry was filtered. The solid was washed with acetone and dried to give title compound, C_11_H_10_BrNO_3_. Single crystals were grown from methanol by the slow evaporation method (yield 0.248 g, 87.32%; m.p.: 441–443 K).

### S3. Refinement

All of the H atoms were placed in their calculated positions and then refined using the riding model with Atom—H lengths of 0.95Å (CH), 0.99Å (CH_2_), 0.88Å (NH) or 0.84Å (OH). Isotropic displacement parameters for these atoms were set to 1.2 (CH, CH_2_, NH) or 1.5 (OH) times *U*_eq_ of the parent atom. The idealised tetrahedral OH was refined as a rotating group: O3A(H3A), O3B(H3B). The highest four peaks in the residual density map are at approximately 1Å from the bromine atoms and have a height of about 2 e^-^/Å^3^.

### Crystal data

**Table:**

C_11_H_10_BrNO_3_	*V* = 1101.38 (11) Å^3^
*M**_r_* = 284.11	*Z* = 4
Triclinic, *P*1	*F*(000) = 568
*a* = 6.2782 (4) Å	*D*_x_ = 1.713 Mg m^−^^3^
*b* = 8.3251 (5) Å	Cu *K*α radiation, λ = 1.54184 Å
*c* = 21.3244 (12) Å	µ = 5.04 mm^−^^1^
α = 96.462 (5)°	*T* = 173 K
β = 92.026 (5)°	Prism, colourless
γ = 95.390 (5)°	0.44 × 0.28 × 0.14 mm

### Data collection

**Table:**

Agilent Eos Gemini diffractometer	3490 reflections with *I* > 2σ(*I*)
Detector resolution: 16.0416 pixels mm^-1^	*R*_int_ = 0.033
ω scans	θ_max_ = 71.3°, θ_min_ = 4.2°
Absorption correction: multi-scan (*CrysAlis PRO* and *CrysAlis RED*; Agilent, 2012)	*h* = −7→6
*T*_min_ = 0.162, *T*_max_ = 1.000	*k* = −8→10
7163 measured reflections	*l* = −26→25
4131 independent reflections	

### Refinement

**Table:**

Refinement on *F*^2^	Primary atom site location: structure-invariant direct methods
Least-squares matrix: full	Hydrogen site location: inferred from neighbouring sites
*R*[*F*^2^ > 2σ(*F*^2^)] = 0.076	H-atom parameters constrained
*wR*(*F*^2^) = 0.227	*w* = 1/[σ^2^(*F*_o_^2^) + (0.1446*P*)^2^ + 3.2341*P*] where *P* = (*F*_o_^2^ + 2*F*_c_^2^)/3
*S* = 1.03	(Δ/σ)_max_ < 0.001
4131 reflections	Δρ_max_ = 2.73 e Å^−^^3^
291 parameters	Δρ_min_ = −0.79 e Å^−^^3^
0 restraints	

### Special details

**Table:**

Geometry. All esds (except the esd in the dihedral angle between two l.s. planes) are estimated using the full covariance matrix. The cell esds are taken into account individually in the estimation of esds in distances, angles and torsion angles; correlations between esds in cell parameters are only used when they are defined by crystal symmetry. An approximate (isotropic) treatment of cell esds is used for estimating esds involving l.s. planes.

### Fractional atomic coordinates and isotropic or equivalent isotropic displacement parameters (Å^2^)

**Table:**

	*x*	*y*	*z*	*U*_iso_*/*U*_eq_	
Br1A	0.19572 (14)	0.90862 (10)	0.46174 (4)	0.0619 (3)	
O1A	0.8488 (6)	0.7490 (4)	0.20741 (18)	0.0363 (9)	
O2A	0.6832 (6)	0.6335 (5)	0.05368 (18)	0.0338 (8)	
O3A	1.0137 (6)	0.7205 (5)	0.02870 (19)	0.0370 (9)	
H3A	0.9464	0.7607	0.0005	0.056*	
N1A	0.5872 (7)	0.5635 (5)	0.2341 (2)	0.0275 (9)	
H1A	0.5246	0.4649	0.2229	0.033*	
C1A	0.7571 (7)	0.6114 (6)	0.2006 (2)	0.0240 (9)	
C2A	0.8300 (8)	0.4766 (6)	0.1541 (2)	0.0266 (10)	
H2AA	0.9036	0.4004	0.1777	0.032*	
H2AB	0.7033	0.4152	0.1307	0.032*	
C3A	0.9795 (8)	0.5448 (6)	0.1078 (2)	0.0274 (10)	
C4A	0.8780 (8)	0.6358 (6)	0.0606 (2)	0.0269 (10)	
C5A	1.1839 (9)	0.5217 (7)	0.1064 (3)	0.0354 (11)	
H5AA	1.2692	0.5636	0.0749	0.042*	
H5AB	1.2461	0.4633	0.1370	0.042*	
C6A	0.5009 (8)	0.6552 (6)	0.2849 (2)	0.0279 (10)	
C7A	0.6134 (10)	0.7860 (7)	0.3221 (3)	0.0375 (12)	
H7A	0.7542	0.8230	0.3118	0.045*	
C8A	0.5228 (11)	0.8617 (8)	0.3733 (3)	0.0448 (14)	
H8A	0.6006	0.9516	0.3981	0.054*	
C9A	0.3196 (10)	0.8086 (7)	0.3892 (3)	0.0400 (13)	
C10A	0.2019 (9)	0.6772 (8)	0.3529 (3)	0.0426 (13)	
H10A	0.0608	0.6415	0.3634	0.051*	
C11A	0.2942 (9)	0.6008 (7)	0.3017 (3)	0.0392 (12)	
H11A	0.2172	0.5099	0.2774	0.047*	
Br1B	1.30729 (11)	0.53869 (9)	0.55597 (3)	0.0518 (3)	
O1B	0.6584 (6)	0.7564 (4)	0.79041 (17)	0.0313 (8)	
O2B	0.8098 (5)	0.8720 (5)	0.94320 (18)	0.0333 (8)	
O3B	0.4808 (6)	0.7820 (5)	0.96889 (19)	0.0365 (8)	
H3B	0.5499	0.7275	0.9919	0.055*	
N1B	0.8836 (7)	0.9562 (5)	0.7586 (2)	0.0310 (9)	
H1B	0.9272	1.0606	0.7645	0.037*	
C1B	0.7305 (7)	0.8992 (6)	0.7954 (2)	0.0237 (9)	
C2B	0.6529 (8)	1.0295 (6)	0.8428 (2)	0.0276 (10)	
H2BA	0.7784	1.0939	0.8657	0.033*	
H2BB	0.5740	1.1040	0.8198	0.033*	
C3B	0.5089 (8)	0.9580 (5)	0.8900 (2)	0.0252 (9)	
C4B	0.6144 (8)	0.8670 (6)	0.9365 (2)	0.0258 (9)	
C5B	0.3032 (8)	0.9786 (7)	0.8930 (2)	0.0331 (11)	
H5BA	0.2219	0.9351	0.9250	0.040*	
H5BB	0.2367	1.0368	0.8632	0.040*	
C6B	0.9792 (8)	0.8566 (6)	0.7107 (2)	0.0297 (10)	
C7B	1.1837 (9)	0.8118 (7)	0.7222 (3)	0.0334 (11)	
H7B	1.2567	0.8449	0.7620	0.040*	
C8B	1.2805 (8)	0.7188 (7)	0.6754 (3)	0.0343 (11)	
H8B	1.4207	0.6889	0.6830	0.041*	
C9B	1.1733 (9)	0.6699 (7)	0.6181 (2)	0.0336 (11)	
C10B	0.9703 (9)	0.7144 (8)	0.6061 (3)	0.0424 (13)	
H10B	0.8977	0.6808	0.5663	0.051*	
C11B	0.8740 (8)	0.8084 (7)	0.6526 (3)	0.0359 (12)	
H11B	0.7350	0.8398	0.6445	0.043*	

### Atomic displacement parameters (Å^2^)

**Table:**

	*U*^11^	*U*^22^	*U*^33^	*U*^12^	*U*^13^	*U*^23^
Br1A	0.0789 (6)	0.0637 (5)	0.0493 (5)	0.0310 (4)	0.0312 (4)	0.0047 (3)
O1A	0.041 (2)	0.0251 (18)	0.040 (2)	−0.0048 (16)	0.0117 (16)	−0.0027 (14)
O2A	0.0245 (18)	0.039 (2)	0.040 (2)	0.0063 (15)	0.0030 (14)	0.0113 (15)
O3A	0.0278 (18)	0.047 (2)	0.039 (2)	0.0037 (16)	0.0037 (15)	0.0125 (16)
N1A	0.027 (2)	0.0222 (19)	0.032 (2)	−0.0017 (16)	0.0045 (16)	−0.0020 (15)
C1A	0.026 (2)	0.020 (2)	0.026 (2)	0.0045 (18)	0.0000 (17)	0.0010 (17)
C2A	0.029 (2)	0.021 (2)	0.030 (2)	0.0065 (18)	0.0011 (18)	0.0014 (17)
C3A	0.028 (2)	0.021 (2)	0.031 (2)	0.0035 (18)	0.0017 (19)	−0.0039 (18)
C4A	0.026 (2)	0.026 (2)	0.027 (2)	0.0023 (18)	0.0053 (18)	−0.0024 (18)
C5A	0.033 (3)	0.040 (3)	0.034 (3)	0.011 (2)	0.001 (2)	0.003 (2)
C6A	0.030 (2)	0.024 (2)	0.032 (2)	0.0059 (19)	0.0064 (19)	0.0058 (18)
C7A	0.044 (3)	0.030 (3)	0.038 (3)	0.000 (2)	0.007 (2)	0.001 (2)
C8A	0.055 (4)	0.040 (3)	0.038 (3)	0.004 (3)	0.007 (3)	−0.001 (2)
C9A	0.047 (3)	0.039 (3)	0.039 (3)	0.019 (3)	0.021 (2)	0.008 (2)
C10A	0.030 (3)	0.046 (3)	0.054 (4)	0.009 (2)	0.016 (2)	0.008 (3)
C11A	0.030 (3)	0.034 (3)	0.054 (3)	0.003 (2)	0.011 (2)	0.002 (2)
Br1B	0.0503 (5)	0.0635 (5)	0.0428 (4)	0.0220 (3)	0.0143 (3)	−0.0059 (3)
O1B	0.0333 (19)	0.0234 (17)	0.0361 (19)	−0.0004 (14)	0.0089 (14)	−0.0006 (14)
O2B	0.0243 (18)	0.038 (2)	0.039 (2)	0.0041 (15)	0.0041 (14)	0.0113 (15)
O3B	0.0252 (17)	0.047 (2)	0.040 (2)	0.0045 (16)	0.0022 (14)	0.0143 (16)
N1B	0.030 (2)	0.024 (2)	0.037 (2)	0.0008 (17)	0.0092 (17)	−0.0013 (16)
C1B	0.020 (2)	0.024 (2)	0.027 (2)	0.0057 (18)	−0.0015 (17)	0.0004 (17)
C2B	0.030 (2)	0.022 (2)	0.032 (2)	0.0050 (19)	0.0047 (19)	0.0008 (18)
C3B	0.026 (2)	0.020 (2)	0.029 (2)	0.0034 (18)	0.0031 (18)	−0.0034 (17)
C4B	0.024 (2)	0.025 (2)	0.028 (2)	0.0052 (18)	0.0026 (17)	−0.0013 (17)
C5B	0.031 (3)	0.037 (3)	0.031 (3)	0.008 (2)	0.002 (2)	−0.001 (2)
C6B	0.028 (2)	0.026 (2)	0.036 (3)	0.0035 (19)	0.0087 (19)	0.0030 (19)
C7B	0.032 (3)	0.035 (3)	0.033 (3)	0.004 (2)	0.002 (2)	0.002 (2)
C8B	0.030 (3)	0.041 (3)	0.034 (3)	0.013 (2)	0.005 (2)	0.004 (2)
C9B	0.033 (3)	0.036 (3)	0.033 (3)	0.008 (2)	0.009 (2)	0.000 (2)
C10B	0.032 (3)	0.060 (4)	0.034 (3)	0.009 (3)	−0.002 (2)	−0.001 (2)
C11B	0.026 (2)	0.046 (3)	0.038 (3)	0.011 (2)	0.002 (2)	0.006 (2)

### Geometric parameters (Å, º)

**Table:**

Br1A—C9A	1.899 (5)	Br1B—C9B	1.893 (5)
O1A—C1A	1.223 (6)	O1B—C1B	1.224 (6)
O2A—C4A	1.225 (6)	O2B—C4B	1.226 (6)
O3A—H3A	0.8400	O3B—H3B	0.8400
O3A—C4A	1.312 (6)	O3B—C4B	1.311 (6)
N1A—H1A	0.8800	N1B—H1B	0.8800
N1A—C1A	1.359 (6)	N1B—C1B	1.346 (6)
N1A—C6A	1.408 (6)	N1B—C6B	1.428 (6)
C1A—C2A	1.527 (6)	C1B—C2B	1.525 (6)
C2A—H2AA	0.9900	C2B—H2BA	0.9900
C2A—H2AB	0.9900	C2B—H2BB	0.9900
C2A—C3A	1.506 (7)	C2B—C3B	1.511 (7)
C3A—C4A	1.487 (7)	C3B—C4B	1.488 (7)
C3A—C5A	1.316 (7)	C3B—C5B	1.321 (7)
C5A—H5AA	0.9500	C5B—H5BA	0.9500
C5A—H5AB	0.9500	C5B—H5BB	0.9500
C6A—C7A	1.391 (7)	C6B—C7B	1.392 (7)
C6A—C11A	1.406 (7)	C6B—C11B	1.382 (8)
C7A—H7A	0.9500	C7B—H7B	0.9500
C7A—C8A	1.367 (8)	C7B—C8B	1.385 (8)
C8A—H8A	0.9500	C8B—H8B	0.9500
C8A—C9A	1.377 (9)	C8B—C9B	1.375 (8)
C9A—C10A	1.400 (9)	C9B—C10B	1.383 (8)
C10A—H10A	0.9500	C10B—H10B	0.9500
C10A—C11A	1.374 (8)	C10B—C11B	1.384 (8)
C11A—H11A	0.9500	C11B—H11B	0.9500
			
C4A—O3A—H3A	109.5	C4B—O3B—H3B	109.5
C1A—N1A—H1A	116.6	C1B—N1B—H1B	118.2
C1A—N1A—C6A	126.8 (4)	C1B—N1B—C6B	123.7 (4)
C6A—N1A—H1A	116.6	C6B—N1B—H1B	118.2
O1A—C1A—N1A	123.8 (4)	O1B—C1B—N1B	123.0 (4)
O1A—C1A—C2A	122.0 (4)	O1B—C1B—C2B	123.2 (4)
N1A—C1A—C2A	114.1 (4)	N1B—C1B—C2B	113.8 (4)
C1A—C2A—H2AA	109.4	C1B—C2B—H2BA	109.2
C1A—C2A—H2AB	109.4	C1B—C2B—H2BB	109.2
H2AA—C2A—H2AB	108.0	H2BA—C2B—H2BB	107.9
C3A—C2A—C1A	111.3 (4)	C3B—C2B—C1B	112.3 (4)
C3A—C2A—H2AA	109.4	C3B—C2B—H2BA	109.2
C3A—C2A—H2AB	109.4	C3B—C2B—H2BB	109.2
C4A—C3A—C2A	115.1 (4)	C4B—C3B—C2B	116.0 (4)
C5A—C3A—C2A	123.7 (5)	C5B—C3B—C2B	123.6 (5)
C5A—C3A—C4A	121.1 (5)	C5B—C3B—C4B	120.3 (5)
O2A—C4A—O3A	123.5 (5)	O2B—C4B—O3B	123.7 (4)
O2A—C4A—C3A	121.9 (5)	O2B—C4B—C3B	122.0 (5)
O3A—C4A—C3A	114.5 (4)	O3B—C4B—C3B	114.2 (4)
C3A—C5A—H5AA	120.0	C3B—C5B—H5BA	120.0
C3A—C5A—H5AB	120.0	C3B—C5B—H5BB	120.0
H5AA—C5A—H5AB	120.0	H5BA—C5B—H5BB	120.0
C7A—C6A—N1A	124.1 (5)	C7B—C6B—N1B	119.2 (5)
C7A—C6A—C11A	118.7 (5)	C11B—C6B—N1B	121.0 (5)
C11A—C6A—N1A	117.0 (5)	C11B—C6B—C7B	119.8 (5)
C6A—C7A—H7A	119.7	C6B—C7B—H7B	120.1
C8A—C7A—C6A	120.5 (6)	C8B—C7B—C6B	119.8 (5)
C8A—C7A—H7A	119.7	C8B—C7B—H7B	120.1
C7A—C8A—H8A	119.7	C7B—C8B—H8B	120.1
C7A—C8A—C9A	120.5 (6)	C9B—C8B—C7B	119.8 (5)
C9A—C8A—H8A	119.7	C9B—C8B—H8B	120.1
C8A—C9A—Br1A	120.8 (5)	C8B—C9B—Br1B	118.8 (4)
C8A—C9A—C10A	120.5 (5)	C8B—C9B—C10B	120.8 (5)
C10A—C9A—Br1A	118.6 (4)	C10B—C9B—Br1B	120.4 (4)
C9A—C10A—H10A	120.6	C9B—C10B—H10B	120.3
C11A—C10A—C9A	118.7 (5)	C9B—C10B—C11B	119.4 (5)
C11A—C10A—H10A	120.6	C11B—C10B—H10B	120.3
C6A—C11A—H11A	119.5	C6B—C11B—C10B	120.3 (5)
C10A—C11A—C6A	121.1 (5)	C6B—C11B—H11B	119.8
C10A—C11A—H11A	119.5	C10B—C11B—H11B	119.8
			
Br1A—C9A—C10A—C11A	−177.6 (5)	Br1B—C9B—C10B—C11B	179.0 (5)
O1A—C1A—C2A—C3A	−15.6 (6)	O1B—C1B—C2B—C3B	10.3 (6)
N1A—C1A—C2A—C3A	166.0 (4)	N1B—C1B—C2B—C3B	−170.7 (4)
N1A—C6A—C7A—C8A	−175.5 (5)	N1B—C6B—C7B—C8B	−178.3 (5)
N1A—C6A—C11A—C10A	176.3 (5)	N1B—C6B—C11B—C10B	178.8 (5)
C1A—N1A—C6A—C7A	−22.1 (8)	C1B—N1B—C6B—C7B	−103.4 (6)
C1A—N1A—C6A—C11A	163.5 (5)	C1B—N1B—C6B—C11B	78.4 (7)
C1A—C2A—C3A—C4A	−70.4 (5)	C1B—C2B—C3B—C4B	69.5 (5)
C1A—C2A—C3A—C5A	112.3 (5)	C1B—C2B—C3B—C5B	−113.4 (5)
C2A—C3A—C4A—O2A	−10.9 (7)	C2B—C3B—C4B—O2B	11.7 (7)
C2A—C3A—C4A—O3A	167.5 (4)	C2B—C3B—C4B—O3B	−168.0 (4)
C5A—C3A—C4A—O2A	166.4 (5)	C5B—C3B—C4B—O2B	−165.5 (5)
C5A—C3A—C4A—O3A	−15.1 (7)	C5B—C3B—C4B—O3B	14.8 (7)
C6A—N1A—C1A—O1A	−6.1 (8)	C6B—N1B—C1B—O1B	−0.6 (8)
C6A—N1A—C1A—C2A	172.3 (4)	C6B—N1B—C1B—C2B	−179.6 (4)
C6A—C7A—C8A—C9A	0.7 (9)	C6B—C7B—C8B—C9B	−0.6 (8)
C7A—C6A—C11A—C10A	1.6 (9)	C7B—C6B—C11B—C10B	0.6 (9)
C7A—C8A—C9A—Br1A	178.0 (5)	C7B—C8B—C9B—Br1B	−178.5 (4)
C7A—C8A—C9A—C10A	−0.6 (10)	C7B—C8B—C9B—C10B	0.9 (9)
C8A—C9A—C10A—C11A	1.0 (9)	C8B—C9B—C10B—C11B	−0.4 (10)
C9A—C10A—C11A—C6A	−1.5 (9)	C9B—C10B—C11B—C6B	−0.3 (9)
C11A—C6A—C7A—C8A	−1.1 (8)	C11B—C6B—C7B—C8B	−0.1 (8)

### Hydrogen-bond geometry (Å, º)

**Table:**

*D*—H···*A*	*D*—H	H···*A*	*D*···*A*	*D*—H···*A*
O3*A*—H3*A*···O2*B*^i^	0.84	1.85	2.685 (5)	174
N1*A*—H1*A*···O1*B*^ii^	0.88	2.06	2.933 (5)	170
O3*B*—H3*B*···O2*A*^iii^	0.84	1.82	2.654 (5)	170
N1*B*—H1*B*···O1*A*^iv^	0.88	2.04	2.848 (6)	152
C5*B*—H5*BB*···O1*A*^v^	0.95	2.54	3.464 (7)	164

Symmetry codes: (i) *x*, *y*, *z*−1; (ii) −*x*+1, −*y*+1, −*z*+1; (iii) *x*, *y*, *z*+1; (iv) −*x*+2, −*y*+2, −*z*+1; (v) −*x*+1, −*y*+2, −*z*+1.

## References

[bb1] Agilent (2012). *CrysAlis PRO* and *CrysAlis RED* Agilent Technologies, Yarnton, England.

[bb2] Allen, F. H., Kennard, O., Watson, D. G., Brammer, L., Orpen, A. G. & Taylor, R. (1987). *J. Chem. Soc. Perkin Trans. 2*, pp. S1–19.

[bb3] Ban, M., Taguchi, H., Katushima, T., Takahashi, M., Shinoda, K., Watanabe, A. & Tominaga, T. (1998). *Bioorg. Med. Chem.* **6**, 1069–1076. 10.1016/s0968-0896(98)00065-0 9730244

[bb4] Dolomanov, O. V., Bourhis, L. J., Gildea, R. J., Howard, J. A. K. & Puschmann, H. (2009). *J. Appl. Cryst.* **42**, 339–341.

[bb5] Galanakis, D., Kourounakis, A. P., Tsiakitzis, K. C., Doulgkeris, C., Rekka, E. A., Gavalas, A., Kravaritou, C., Christos, C. & Kourounakis, P. N. (2004). *Bioorg. Med. Chem. Lett.* **14**, 3639–3643. 10.1016/j.bmcl.2004.05.025 15203134

[bb6] Gududuru, V., Hurh, E., Dalton, J. T. & Miller, D. D. (2004). *Bioorg. Med. Chem. Lett.* **14**, 5289–5293. 10.1016/j.bmcl.2004.08.029 15454213

[bb7] Kumar, P. & Knaus, E. E. (1993). *Eur. J. Med. Chem.* **28**, 881–885.

[bb8] Lesyk, R. & Zimenkovsky, B. (2004). *Curr. Org. Chem.* **8**, 1547–1578.

[bb9] Nayak, P. S., Narayana, B., Jasinski, J. P., Yathirajan, H. S. & Kaur, M. (2013*b*). *Acta Cryst.* E**69**, o1752. 10.1107/S160053681302998X PMC3885031 24454206

[bb10] Nayak, P. S., Narayana, B., Yathirajan, H. S., Gerber, T., Brecht, B. van & Betz, R. (2013a). *Acta Cryst.* E**69**, o83. 10.1107/S160053681205012X PMC3588263 23476465

[bb11] Palatinus, L., Prathapa, S. J. & van Smaalen, S. (2012). *J. Appl. Cryst.* **45**, 575–580.

[bb12] Sheldrick, G. M. (2008). *Acta Cryst.* A**64**, 112–122. 10.1107/S0108767307043930 18156677

[bb13] Ukrainets, I. V., Sidorenko, L. V., Petrushovo, L. A. & Gorokhova, O. V. (2006). *Chem. Heterocycl. Comput.* **42**, 64–69.

